# Structural-Functional Analysis of 2,1,3-Benzoxadiazoles and Their N-oxides As HIV-1 Integrase Inhibitors

**Published:** 2013

**Authors:** S. P. Korolev, O. V. Kondrashina, D. S. Druzhilovsky, A. M. Starosotnikov, M. D. Dutov, M. A. Bastrakov, I. L. Dalinger, D. A. Filimonov, S. A. Shevelev, V. V. Poroikov, Y. Y. Agapkina, M. B. Gottikh

**Affiliations:** Department of Chemistry, Lomonosov Moscow State University, Leninskie gory, 1/3, Moscow, Russia, 119991; Belozersky Research Institute of Physicochemical Biology, Lomonosov Moscow State University, Leniskie gory, 1/40, Moscow, Russia, 119991; Orekhovich Institute of Biomedical Chemistry, Russian Academy of Medical Sciences, Pogodinskaya Str., 10/8, Moscow, Russia, 119121; Zelinsky Institute of Organic Chemistry, Russian Academy of Science, Leninskiy prospekt, 47, Moscow, Russia, 119991

**Keywords:** HIV-1 integrase, inhibition, nitrobenzofuroxan, nitrobenzofurazan, PASS, QikProp

## Abstract

Human immunodeficiency virus type 1 integrase is one of the most attractive targets for the development of anti-HIV-1 inhibitors. The capacity of a series of 2,1,3-benzoxadiazoles (benzofurazans) and their N-oxides (benzofuroxans) selected using the PASS software to inhibit the catalytic activity of HIV-1 integrase was studied in the present work. Only the nitro-derivatives of these compounds were found to display inhibitory activity. The study of the mechanism of inhibition by nitro-benzofurazans/benzofuroxans showed that they impede the substrate DNA binding at the integrase active site. These inhibitors were also active against integrase mutants resistant to raltegravir, which is the first HIV-1 integrase inhibitor approved for clinical use. The comparison of computer-aided estimations of the pharmacodynamic and pharmacokinetic properties of the compounds studied and raltegravir led us to conclude that these compounds show promise and need to be further studied as potential HIV-1 integrase inhibitors.

## INTRODUCTION

The human immunodeficiency virus (HIV) is responsible for the acquired immunodeficiency syndrome (AIDS), which is one of the most dangerous diseases. The extremely high rates of growth in the number of HIV-infected patients in Russia make the development of effective medical therapies to combat the virus a particularly pressing challenge for the country. The viral enzyme integrase (IN), catalyzing the integration of viral DNA into cellular DNA, which is the key stage in the replication cycle of HIV, is considered to be one of the most promising targets for HIV-1 inhibitors [1].

Highly active antiretroviral therapy is used to treat HIV infections and currently includes 25 drugs [2]; most of them inhibit two viral enzymes: reverse transcriptase and protease. In late 2007, the first IN inhibitor (IsentressTM, or Raltegravir) was approved for use as a new agent for AIDS therapy [3]. However, even combination therapy cannot fully suppress viral replication, and the virus develops resistance to drugs over time. It is now known that resistance to Raltegravir develops in some patients within 12 weeks [4]. The majority of IN inhibitors currently at the stage of clinical trials are similar to Raltegravir in terms of their mechanism of action [5]. Raltegravir-induced cross-resistance to these compounds has already been demonstrated to develop in patients [6]. Thus, designing new integration inhibitors that would differ from Raltegravir in terms of their mechanism of action is currently a pressing need.

Computer-aided design methods are now widely used to search for new physiologically active substances and optimize their structure [7]. In particular, computer-aided methods are used to design HIV-1 IN inhibitors [8–10]. The PASS computer program developed by us [11, 12] was used to perform virtual screening and selection of potential IN inhibitors among commercially available and potentially synthesizable compounds [13, 14]. The derivatives of 2,1,3 -benzoxadiazoles (benzofurazans) and their N-oxides (benzofuroxans) were selected using a specialized version of the PASS program [14]. These compounds have been synthesized; their ability to inhibit the catalytic activity of HIV-1 IN was experimentally tested in the present study.

Two reactions are catalyzed by IN during viral replication: the 3’-end processing of viral DNA, resulting in the removal of the dinucleotides GT from both 3’-ends; and the strand transfer reaction, during which viral DNA is incorporated into cellular DNA. Raltegravir and its analogs are known as strand transfer inhibitors, since they suppress this particular reaction more effectively [15]. Benzofurazan (BFZ) and benzofuroxan (BFX) were found to generally exert the same effects on both reactions catalyzed by IN. It was demonstrated that the inhibitory effect of these compounds is highly dependent on the presence of a nitro group. Among a series of substituted 4-nitro-BFZ/BFX, certain compounds capable of blocking IN at a concentration of 0.5–1 μM have been identified. These inhibitors were found to be also active against Raltegravir-resistant IN mutants.

The pharmacodynamic and pharmacokinetic characteristics of BFZ and BFX were assessed using the PASS and QikProp computer programs [16]. The potential benefits of these compounds as compared to those of Raltegravir were demonstrated.

## EXPERIMENTAL

**Computer programs and databases**

A specialized version of the computer program PASS, trained on a sample of 218 compounds with the determined IN suppressive capabilities, was used for virtual screening of the databases of commercially available samples and potentially synthesizable compounds to select substances that are likely to inhibit HIV-1 IN [14]. Thirty-five of these compounds affect the 3’-processing (IC_50_ < 100 μM), Twenty-eight of them inhibit the strand transfer reaction (IC_50_ <100 μM), the remaining compounds exhibit no inhibitory properties. The overall pharmacological profile of the new IN inhibitors was evaluated using the contemporary standard version of PASS (12.06.22) [11, 12], which allows one to predict 513 possible toxic and side effects. The result is given to the user as an ordered list of possible biological activities with the estimated Pa and Pi, which characterize the probability of presence/absence of each type of activity, respectively.

QikProp was used to assess the ADME pharmacokinetic parameters of the analyzed molecules [16]. The program enables to assess the physical and chemical characteristics of the drug similarity and is commonly used to screen compounds with undesired pharmacokinetic characteristics [17–19]. The range of parameter values determined by QikProp and recommended for the promising compounds was provided in [14].

**1,2,5-benzoxadiazols (benzofurazans) and their N-oxides (benzofuroxans)**

1,2,5-benzoxadiazols and their N-oxides were synthesized using the conventional [20–22] or analogous procedures.

**Oligodeoxyribonucleotides**

Oligodeoxyribonucleotides were synthesized using the amidophosphite method on an ABI 3400 automated DNA synthesizer (Applied Biosystems, USA) in accordance with the standard operating procedures using commercially available reagents (Glen Research, USA). Oligonucleotides U5B (5’-GTGTGGAAAATCTCT AGCAGT-3’) and U5A (5’-ACT GCT AGAGATTTTC ACAC-3’) formed a duplex imitating the end fragment of the U5-moiety of the long terminal repeat of viral DNA, which acts as a substrate for IN during the 3’-processing reaction. The duplex formed from the oligonucleotides U5B-2 (5’-GTGTGGAAAATCTCT AGCA-3’) and U5A was used in the chain transfer reaction. The effects of the inhibitors on correct DNA folding at the IN active site was assessed using the U5B/U5A^m^’ duplex (5’- ACT ^m^’GCT AGAGATTTTC ACAC-3’), where T^m^’ was 2’-O-(2,3-dihydroxypropyl)-uridine synthesized according to [23]. The N155H (5’-CT - GTCCT ATAATTTTCTTT AATTCTTT ATGCATAGATTCT ATT ACCCCCT GA-3’), G140S (5’-GGGGATCAAGCAGGAATTT AGCATTCCCT ACAATC -3’), Q148K (5’-GCATTCCCT ACAATCCCC AAAGTAAGGGGGTAATAG- 3’) oligonucleotides and their complementary N155H_a, G140S_a, and Q148K_a oligonucleotides were used as primers for site-directed mutagenesis of the HIV-1 integrase gene to produce mutant forms of the integrase gene (N155H, G140S/ Q148K).

**Enzymes**

The recombinant HIV-1 IN was isolated from the cells of the Rosetta *Escherichia coli *producer strain and purified without the addition of a detergent as per [24]. The plasmids containing the mutant forms of the IN genes (N155H and G140S/Q148H substitutions) were obtained by site-directed mutagenesis of a plasmid encoding wild-type IN using the QuikChange II Site-Directed Mutagenesis kit (Agilent Technologies, USA). All procedures were performed in accordance with the manufacturer’s instructions. Mutant proteins were isolated and purified as per wild-type of HIV-1 IN [24].

**Synthesis of ^32^P-labeled integrase substrate**

Radioactive ^32^P label was introduced into the 5’-end of the oligonucleotide U5B or U5B-2. To achieve this, 10 pmol of the oligonucleotide was incubated in the presence of T4-polynucleotide kinase (Fermentas, Lithuania) and 50 μCi [γ-^32^P]ATP (3000 Ci/mmol) in a buffer containing 50 mM Tris-HCl, pH 7.5, 10 mM MgCl_2_, 5 mM dithiothreitol (DTT ), 0.1 mM spermidine, 0.1 mM EDTA, for 1 h at 37°C. Following this procedure, the kinase was inactivated by adding EDTA (25 mM) and heating to 65°C for 10 min. An equimolar amount of the complementary oligonucleotide, U5A, was added, and a duplex was formed by heating the oligonucleotide mixture to 95°C followed by slow cooling to room temperature. The U5B/U5A duplex was completely purified of excess [γ-^32^P]ATP and salts on a MicroSpin G-25 column (Amersham Biosciences, USA).

**Inhibition of the 3’-end processing reaction**

The ^32^P-labeled U5B/U5A duplex (3 nM) was incubated in 20 μl of the buffer (20 mM HEPES, pH 7.2, 7.5 mM MgCl_2_, 1 mM DTT ) in the presence of IN (100 nM) and increasing concentrations of the inhibitor at 37°C for 2 h. The reaction was stopped using 80 μl of a stop solution (7 mM EDTA, 0.3 M NaOAc, 10 mM Tris-HCl, pH 8, 0.125 mg/ml glycogen). The integrase was extracted using phenol-chloroform-isoamyl alcohol = 25 : 24 : 1; the DNA-duplex was precipitated with ethanol (250 μl) and assayed by 20% polyacrylamide gel electrophoresis (PAGE) with 7 M urea. The gel was visualized in a STORM 840^TM^ Phosphorimager (Molecular Dynamics, USA). The reaction was recorded according to the band in electrophoretic pattern, which corresponded in terms of its mobility to oligonucleotide U5B truncated by two residues. The reaction efficiency was assessed using the Image QuaNT^TM^ 4.1 program. The results of three independent repetitions of the experiment were used to build a curve representing the relationship between the efficiency of 3’-processing and the inhibitor concentration. The curve was used to identify the value of IC_50_ as the inhibitor concentration at which the reaction is suppressed to 50%.

**Inhibition of the strand transfer reaction**

The reaction was carried out as per inhibition of the 3’-processing using the ^32^P-labeled U5B-2/U5A duplex (10 nM) and IN (100 nM). The reaction was recorded according to the bands in the electrophoretic pattern with a lower mobility as compared to that of the initial oligonucleotide, U5B-2.

**Gel shift analysis**

The ^32^P-labeled U5B/U5A duplex (0.05 pmol) was incubated in the presence of integrase (2 pmol) in a buffer containing 20 mM HEPES, pH 7.2, 7.5 mM MgCl_2_, 1 mM DTT , 5% glycerol at 20°C for 30 min. Increasing amounts of the oligonucleotide inhibitor (0.01–10.0 μM) were added to the preformed enzyme-substrate complex; the mixture was incubated at 37°C for 5 min and then applied to an 8% polyacrylamide gel (acrylamide/ bisacrylamide ratio = 40: 1) with no urea. The electrophoresis buffer contained 20 mM Tris-acetate, pH 7.2, 7.5 mM MgCl_2_. The gel was visualized with a STORM 840^TM^ Phosphorimager.

**The effects of the inhibitor on the correct folding of DNA at the IN active site**

The 2,3-dihydroxypropyl group consisting of the oligonucleotide duplex was oxidized to an aldehyde group immediately prior the experiment: 15 μl of a freshly prepared 230 mM aqueous solution of sodium periodate was added to 10 pmol of the U5B/U5A^m^’ duplex containing the ^32^P-labeled modified oligonucleotide U5A^m^’ in 15 μl of 30 mM sodium acetate (pH 4.5). The mixture was stirred and incubated for 1 h at 25°C in the dark followed by the addition of 170 μl of a 2 M aqueous solution of lithium perchlorate; the oligonucleotide material was precipitated using 1 ml of acetone. The obtained U5B/U5A^m^ duplex containing the oligonucleotide with a 2’-aldehyde group (U5A^m^) was dissolved in a buffer containing 20 mM HEPES, pH 7.2, 7.5 mM MgCl_2_, 1 mM DTT . Covalent attachment of the oxidized U5B/U5A^m^ duplex (10 nM) to the IN (100 nm) was carried out in 20 μl of a buffer containing 20 mM HEPES, pH 7.2, 7.5 mM MgCl_2_, 1 mM DTT in the presence of increasing concentrations of the inhibitor for 1 h at 37°C . The reaction product was then reduced by adding 2 μl of a freshly prepared 300 mM solution of NaBH_3_CN and incubating for 30 min at 37°C. The reaction mixture was analyzed in the Laemmli PAGE system. The labeled products were visualized using the STORM 840^TM^ Phosphorimager. The efficiency of the reaction progress was assessed by observing the intensity of the band corresponding to the covalently bound IN-DNA complex using Image QuaNT^TM^ 4.1.

## RESULTS AND DISCUSSION

**Computer-aided screening of new integrase inhibitors**

A specialized version of the PASS program was used for computer-aided screening and selection of the substances that are highly likely to possess HIV-1 IN inhibitory potential [14]. The accuracy of anti-integrase activity prediction calculated for the training set of 218 compounds using the leave-one-ROI-out technique was 81%. The biological activity of Raltegravir, which effectively inhibits the chain transfer reaction, was predicted using this particular version of PASS (*Table 1*). The estimated probability of exhibiting this activity was 0.948 for Raltegravir (Pa). These facts suggest that PASS has a significant capability of predicting the anti-integrase activity of compounds.

Over two millions structural formulas of substances belonging to various chemical classes were analyzed. The structures selected using the computer-aided predictions turned out to be the derivatives of benzofurazans and benzofuroxans. In order to perform structural- functional studies, 27 different compounds belonging to these structural classes characterized by an estimated probability of possessing the ability to inhibit the 3’-processing and chain transfer reactions greater than 0.5 were synthesized (*Table 1*).

**The effects of the structure of 1,2,5-benzoxadiazols on their ability to inhibit IN activity**

The ability of BFZ and BFX to suppress the catalytic activity of IN was investigated in the 3’-end processing and strand transfer reactions using recombinant protein and U5B/U5A and U5B-2/U5A DNA-duplexes corresponding to the terminal fragment of viral DNA prior to and following the cleavage of the GT dinucleotide. The U5B/U5A duplex acted as the IN substrate in the 3’-end processing reaction, while the U5B-2/U5A duplex acted as the IN substrate in the strand transfer. It should be noted that the recombinant IN can use any DNA as a target for incorporation of the processed substrate in the strand transfer reaction; hence, the U5B-2/U5A duplex acted both as a substrate and as a target in this reaction.

The unsubstituted BFX exhibited no inhibitory potential in any of the reactions (*Table 1*, compound **1**). The introduction of an electron-donor (methyl) or electron- acceptor (chlorine) substituent at the 5-position only insignificantly improved the inhibitory activity of BFX during the strand transfer (*Table 1* **, 2 **and **3**). However, 4-nitro-BFX was a significantly more efficient inhibitor in both reactions (*Table 1* **, 4**).

With allowance for such a strong influence of the nitro group, the effects of the substituents at positions 5 and 7 on the activity of 4-nitro-BFX were examined. It was demonstrated that the presence of a methyl residue at any of these positions significantly increases the inhibitory activity of 4-nitro-BFX (*Table 1*, **5 **and** 6**). In this case, the presence of a methyl group at both positions had no additional positive effect, but instead slightly reduced the inhibition efficiency (*Table 1*, **7**). The enhancement of the inhibitory activity of 4-nitro- BFX after a methyl group was introduced at position 5 or 7 can be attributed to the electron donor effect of a methyl group. To support or refute this assumption, the efficiency of inhibition of processing and strand transfer by 4-nitro-BFX containing other electrondonating substituents at position 7 was assessed (*Table 1*, **8 **and **9**). Both of these compounds were found to block IN with an efficiency comparable to that of the unsubstituted 4-nitro-BFX. Thus, the positive inductive effect of the methyl group cannot be the reason for the increased inhibitory activity of compounds **5 **and **6**. The ability of the methyl group to form hydrophobic interactions with the protein is also an unlikely reason for the observed effect, since the methoxy group is also theoretically capable of these interactions. It is interesting that the derivative of 4-nitro-BFX containing an electron-acceptor substituent in the form of chlorine at position 7 was a more potent inhibitor as compared to the original 4-nitro-BFX (*Table 1*, **10**) but was inferior to 7-methyl-4-nitro-BFX in terms of its inhibitory properties.

Next, the importance of the role of N-oxide was determined. For this purpose, the inhibitory effect of 4-nitro-BFX derivatives and the corresponding derivatives of 4-nitro-BFZ were compared. Unsubstituted 4-nitro-BFZ inhibited both of the reactions under study to a greater extent than N-oxide did (*Table 1*, **11** and **4**). However, its methyl derivatives (compounds **12** and **13**) were 3–6 times less active than compounds **5** and **6 **(*Table 1*). Nevertheless, the patterns observed for the 4-nitro-BFX derivatives were generally valid for a series of 4-nitro-BFZ derivatives (*Table 1*).

Thus, it can be concluded that 4-nitro-BFZ derivatives and the corresponding N-oxides are capable of blocking HIV-1 IN with comparable efficiencies; the level of efficiency depends on the nature of the substituents at position 5 or 7. Methyl-substituted 4-nitro- BFZ and 4-nitro-BFX were found to be the most efficient inhibitors.

**Table 1:** The ability of BFZ and BFX derivatives to inhibit the IN catalytic activity during 3’-processing and strand transfer reactions

Structure	No	R1	R2	Inhibitory activity, IC_50_, μM*	
				3’-processing	strand transfer
Raltegravir				0.50 ± 0.09	0.010 ± 0.003
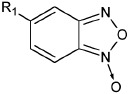	1	H	-	> 1000	> 1000
	2	CH_3_	-	> 1000	800 ± 200
	3	Cl		> 1000	500 ± 200
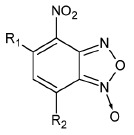	4	H	H	80 ± 20	80 ± 30
	5	CH_3_	H	0.4 ± 0.1	1.0 ± 0.3
	6	H	CH_3_	0.5 ± 0.2	0.4 ± 0.2
	7	CH_3_	CH_3_	1.0 ± 0.3	7 ± 2
	8	H	OCH_3_	70 ± 20	80 ± 20
	9	H	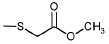	50 ± 10	80 ± 30
	10	H	Cl	20 ± 5	50 ± 10
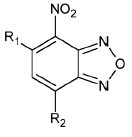	11	H	H	30 ± 5	40 ± 10
	12	CH_3_	H	2.0 ± 0.4	3.0 ± 0.6
	13	H	CH_3_	3.0 ± 0.6	3.0 ± 0.5
	14	OCH_3_	H	75 ± 12	150 ± 40
	15	H	OCH_3_	80 ± 30	120 ± 20
	16	H	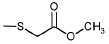	65 ± 11	70 ± 20
	17	H	Cl	10 ± 2	45 ± 12
	18	H	-SO_2_-Ph	20 ± 5	15 ± 5
	19	H	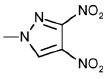	10 ± 2	12 ± 3
	20	H	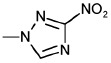	18 ± 6	20 ± 5
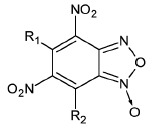	21	H	H	400 ± 100	500 ± 120
	22	H	CH_3_	2.0 ± 0.4	0.3 ± 0.1
	23	H	CH_2_Br	6 ± 2	2.0 ± 0.5
	24	H	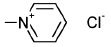	75 ± 15	80 ± 20
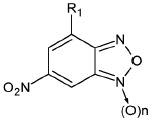	25	H	-	0.5 ± 0.1	5 ± 2
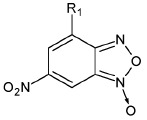	26	H	-	6 ± 1	5 ± 1
	27	CH_3_	-	100 ± 20	100 ± 30

* The average values calculated from the results of at least three repeated experiments.

**Table 2:** The inhibition of the catalytic activity of the Raltegravir-resistant mutant forms of IN by the nitro-BFX/BFZ derivatives

Compound, N^o^	Inhibitory activity during the strand transfer reaction, IC_50_, μM*					
	wild-type		Q148K/G140S mutant		N155H mutant	
	IC_50_	IC_95_	IC_50_	IC_95_	IC_50_	IC_95_
Raltegravir	0.010 ± 0.003	0.40 ± 0.05	0.15 ± 0.03	3.0 ± 1.0	0.018 ± 0.005	5.2 ± 0.8
6	0.4 ± 0.2	3.5 ± 0.9	0.8 ± 0.3	4.3 ± 0.8	0.9 ± 0.3	8.2 ± 1.7
22	0.3 ± 0.1	6.8 ± 1.1	0.5 ± 0.2	1.0 ± 0.4	0.6 ± 0.1	7.6 ± 1.3
25	5.0 ± 2.0	18.0 ± 2.0	4.0 ± 1.5	15.3 ± 2.8	6.0 ± 1.8	18.8 ± 2.5

* The average values calculated from the results of at least three repeated experiments.

In addition to BFX containing a single nitro group, the derivatives of 4,6-dinitro-BFX were also tested as IN inhibitors (*Table 1*, **21–24**). It was found that the introduction of the second nitro group significantly reduced the inhibitory activity (compare **21 **and **4**). However, the presence of a methyl substituent at position 7 in the case of 4,6-dinitro-BFX also significantly increased the efficiency of inhibition of integration and strand transfer (the latter process was inhibited 6–7 times more efficiently than 3’-processing) (*Table 1*, **22**). It is interesting to point out that compound **23 **containing an electron-accepting bromomethyl substituent also effectively inhibited both reactions, although it was somewhat inferior to 7-methyl-4,6-dinitro-BFX (**22**) in this respect. Meanwhile, compound **24**, which contains a very strong electron acceptor at position 7, exhibited a low inhibitory activity (*Table 1*).

The integration inhibition properties of 6-nitro-BFX and 6-nitro-BFZ were subsequently studied (*Table 1*, **25** and **26**). Both compounds were found to be significantly more efficient in inhibiting the integration process as compared to 4-nitro-BFX/BFZ and 4,6-dinitro-BFX (*Table 1*, **4, 11, 21**). It is of little interest that the effects of 6-nitro-BFX and 6-nitro-BFZ in the chain transfer reaction were identical, and the 3’-processing was more efficiently inhibited by 6-nitro-BFZ (**25**). The introduction of the methoxy group at position 4 significantly reduced the inhibition efficiency (*Table 1*, **27**).

The ability of BFX and BFZ containing nitro groups at positions 4 and/or 6 to inhibit both reactions catalyzed by HIV-1 IN with almost identical efficiency gave grounds to assume that the mechanism of integration inhibition used by these compounds differs from the mechanism of action of Raltegravir, which mainly inhibits the stand transfer [15]. In order to verify this hypothesis, the potential of nitro-BFX/BFZ to inhibit the mutant Raltegarvir- resistant forms of IN was evaluated.

**Inhibition of the mutant forms of IN characterized by increased resistance to Raltegravir**

The emergence of resistance to strand transfer inhibitors is attributable to the emergence of mutations at the IN active site [25]. Patients with Raltegravir resistance typically carry primary mutations, such as Y143R/C, Q148K/R/H, and N155H. Q148R/H/K and N155H amino acid substitutions are also common in patients treated with IN inhibitor Elvitegravir, which has just been approved by the FDA for clinical use in HIV therapy [26]. For this reason, IN proteins containing Q148K and N155N substitutions were used in the present work. With allowance for the fact that the replacement of Q148 dramatically decreases the IN activity, which is reduced due to a secondary mutation in the G140 residue [27], an IN specimen containing the double mutation G140S/Q148K was obtained. The ability of the most active compounds **6**, **22 **and **25**, which represented all three investigated groups of nitro-BFX/BFZ, to inhibit the catalytic activity of the mutant proteins and wildtype IN during the strand transfer reaction was tested. It was found that the inhibitors analyzed suppress the activity of all IN specimens with comparable efficiencies (*Table 2*). Meanwhile, the two mutant forms of IN were inhibited by Raltegravir to a lower extent than the wildtype IN; the reduction in the inhibition efficiency was particularly evident for the IC_95_ values (*Table 2*).

**Investigation of the inhibitory mechanism of nitro-BFX/BFZ derivatives**

The ability of nitro-BFX/BFZ derivatives to inhibit the mutant forms of IN as efficiently as the wild-type enzyme confirmed the validity of the assumption that the mechanism of integration inhibition by these compounds differs from that of Raltegravir. In order to better understand the inhibitory mechanism of nitro- BFX/BFZ derivatives, the compounds were selected according to two criteria: 1) compounds were selected from all three groups of the derivatives differing by the position and number of nitro groups and 2) compounds with different substituents were selected, since the formation of additional contacts between the protein and the substituent could potentially influence the inhibitory mechanism. Therefore, compounds **6**, **9 **and **18 **were selected from the 4-nitro-BFX/BFZ group; compound** 23**, from the group of 4,6-dinitro-BFX, and compound** 25**, from the group of 5-nitro-BFX/BFZ (*Table 1*).

It should be mentioned that all of the strand transfer inhibitors act through the same mechanism: they bind to the active site of IN, which forms a complex with the viral DNA and prevent its interaction with cellular DNA [5, 15, 28]. The compounds equally efficient at inhibiting both stages of integration can have different mechanisms of action. They can either interact with the C-terminal domain disrupting the binding of DNA, or they can bind to the catalytic domain of IN affecting or not affecting the correct folding of viral DNA, or they can interact with the other parts of IN; e.g., acting as allosteric inhibitors [5, 29].

Initially, the effects of inhibitors on the DNA-binding activity of IN were studied. The C-terminal domain of IN is mainly responsible for the binding to DNA [30]. Therefore, the inhibitor that suppresses both DNA binding and 3’-processing when taken at equal concentrations affects the C-terminal domain. The action of the inhibitors on the DNA binding was studied at 25°C, since IN completely binds to the DNA-substrate under these conditions to form an enzyme-substrate complex, but does not perform a catalytic function [31]. It was found that almost all the investigated compounds affect DNA binding to IN to a much lesser extent than they affect the 3’-processing (*Table 3*, columns 5 and 1). This fact led to the assumption that the inhibitors interact with the catalytic domain of IN.

**Table 3:** The effects of nitro-BFX and nitro-BFZ on the catalytic activity of IN in 3’-processing and strand transfer reactions, and on the IN DNA-binding activity and binding of the DNA-substrate at the active site of IN

Compound, N^o^	Inhibitory activity during the strand transfer reaction, IC, μM*					
	3’-processing		strand transfer		binding of IN to DNA	binding of DNA at the active site of IN
	Mg^2+^	Mn^2+^	Mg^2+^	Mn^2+^		
	1	2	3	4	5	6
Raltegravir	0.50 ± 0.09	0.15 ± 0.02	0.010 ± 0.003	0.005 ± 0.002	> 500	> 500
6	0.5 ± 0.2	0.5 ± 0.1	0.4 ± 0.2	0.5 ± 0.2	10 ± 2	0.6 ± 0.2
9	50 ± 10	35 ± 10	80 ± 30	70 ± 20	500 ± 100	90 ± 20
18	20 ± 5	20 ± 5	15 ± 5	25 ± 5	50 ± 10	20 ± 5
23	6 ± 2	5 ± 2	2.0 ± 0.5	5 ± 2	25 ± 8	6 ± 2
25	0.5 ± 0.1	1.0 ± 0.2	5 ± 2	4 ± 1	45 ± 10	1.0 ± 0.5

* The average values calculated from the results of at least three repeated experiments.

An inhibitor binding to the catalytic domain of IN can prevent “correct” interaction between the viral DNA and the active site of the enzyme without affecting the overall DNA–IN binding. The influence of the inhibitors on the correct folding of DNA-substrate at the active site of IN was studied using the method of covalent attachment of the aldehyde-containing analog of DNA-substrate to IN [32]. The aldehyde group was introduced into the structure of the modified thymidine analogue (T^m^), which occupied position 3 counting from the 5’-end of oligonucleotide U5A (*Figure, A*), since it was located close to the amino acid residues of the IN catalytic domain [33]. The feasibility of this approach was described in [34, 35].

The U5B/U5A^m^ duplex containing a radioactive label in the U5A^m^ chain was covalently attached to the IN in the presence of increasing concentrations of inhibitors; the influence of inhibitors on the efficiency of the reaction was analyzed (*Figure, B*). No inhibition of the covalent attachment was observed in the case of Raltegravir (*Table 3*, column 6), which is consistent with the findings that strand transfer inhibitors do not affect the interaction between IN and viral DNA [5, 15]. The IC_50_ values for all nitro-BFX/BFZ derivatives for the inhibition of the covalent DNA binding at the IN active site were similar to those obtained for the catalysis (*Table 3*, columns 6 and 1). This fact indicates that the inhibitors interact with the active site of IN and prevent the correct folding of the DNA–substrate within it. However, the binding of inhibitors does not cause such changes in the IN structure that can completely block its DNA binding activity.

Under the assumption that the derivatives of nitro- BFX/BFZ bind at the active site of IN, we decided to clarify whether they interact with the metal-cofactor ions, which are bound at the IN active site and are essential for its catalytic activity [36]. Mg^2+^ is a native cofactor of IN, but *in vitro *IN efficiently catalyzes both reactions in the presence of Mn^2+^ ions as well. If the inhibitor interacts with the metal ion, its effects on the IN activity in the presence of these metal ions will differ due to the differences in the coordinating ability of Mg^2+^ and Mn^2+^ ions. This very effect was observed for Raltegravir (*Table 3*, columns 1–4). The results of inhibition of 3’-processing and the strand transfer reaction in the presence of various metal ions demonstrate that the type of the metal does not affect the efficiency of the action of nitro-BFX/BFZ derivatives. It is obvious that the interaction between these inhibitors and the active site of IN is not mediated by binding to the metal ion.

**Prediction on the pharmacodynamic and pharmacokinetic characteristics of nitro-BFX/BFZ derivatives**

**Table 4:** The spectra of potential toxicity/side effects of nitro-BFZ and nitro-BFX as compared to Raltegravir

Compound, N^o^	Predicted toxic and side effects (Pa > 0.5)		
	Pa*	Pi*	Activity
6	0.536	0.068	Hypotension
	0.503	0.085	Vessel toxicity
9	-	-	-
18	0.595	0.015	Carcinogenicity (rats, males, kidneys)
	0.551	0.014	Carcinogenicity (rats, males)
	0.519	0.020	Stimulator of tear secretion
23	0.653	0.005	Mutagenic
	0.556	0.006	Mutagenic
25	0.816	0.014	Vessel toxicity
	0.679	0.007	Carcinogenicity (rats, males)
	0.661	0.008	Carcinogenicity (rats, females)
	0.632	0.019	QT-interval prolongation
	0.571	0.013	Carcinogenicity (rats, females, mammary gland)
	0.603	0.049	Hypotension
	0.588	0.034	Allergic dermatitis
	0.583	0.047	Cyanosis
	0.570	0.045	Ototoxicity
	0.568	0.074	Hemotoxicity
Raltegravir	0.933	0.003	Hyperkinesia
	0.932	0.004	Ataxia
	0.923	0.004	Anxiety
	0.861	0.013	Vertigo
	0.850	0.010	Thrombocytopenia
	0.830	0.017	Sensory impairments
	0.796	0.023	Vomiting
	0.780	0.016	Dyskinesia
	0.787	0.025	Dermatitis
	0.783	0.022	Headache
	0.781	0.024	Allergic reaction
	0.744	0.031	Pain
	0.702	0.040	Nausea
	0.683	0.032	Nephrotoxicity
	0.693	0.042	Sleeping disorders
	0.603	0.065	Hemotoxicity
	0.589	0.073	Gastro-intestinal toxicity
	0.556	0.065	Hepatotoxicity

* Pa – probability of observing activity; Pi – probability of observing inactivity

The standard version of the PASS program (version 12.06.22) was used to predict the possible toxic and side effects of compounds **6, 9, 18, 23, 25**, and Raltegravir (*Table 4*). It should be mentioned that 15 out of 18 predicted (Pa > 0.5) toxic and side effects of Raltegravir correspond to the data obtained during experimental and clinical studies [37]. No side/toxic effects have been identified in the predicted spectra of biological activity of compound** 9**. Compounds **6, 18, 23 **and **25 **can cause some undesirable side effects, although it should be borne in mind that the side effects predicted by PASS may occur at concentrations exceeding therapeutic doses.

The calculation of the ADME characteristics using QikProp showed that all 18 parameters of compounds **6, 9, 18, 23 **and **25 **correspond to the recommended range [13]. The estimated IC_50_ for blockage of HER G K^+^ channels is obtained from this range for Raltegravir (less than –5) [14]. This corresponds to the data obtained in [38], according to which Raltegravir at high concentrations acts as a blocker of HER G К^+^ channels, which can result in prolongation of the QT-interval and, consequently, in the development of heart failure.

## CONCLUSIONS

**Figure:**
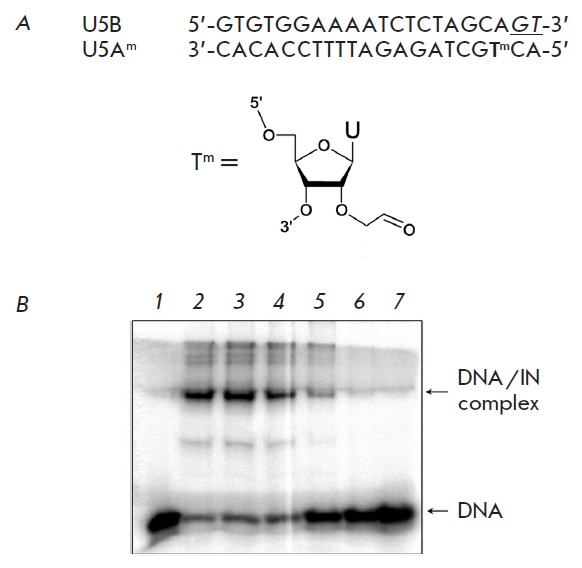
The influence of compound **6 **on the efficiency of covalent binding of a DNA-substrate analog containing the aldehyde group to IN. *A *– the structure of the U5B/U5A^m^ DNA-substrate analog and modified thymidine Т^m^. GT dinucleotide cleaved by IN during the 3’-processing is underlined and shown in italics. *B *– the analysis of the influence of inhibitor** 6 **on the covalent binding of the U5B/U5A^m^ duplex to IN using Laemmli gel-electrophoresis. Lanes: *1 *– control; *2 *– 0 μM of **6**; *3 *– 0.1 μM of **6**; *4 *– 0.5 μM of **6**; *5 *– 1 μM of **6**;* 6 *– 10 μM of **6**; *7 *– 100 μM of **6**

Thus, the new class of IN inhibitors identified using computer prediction, nitro-BFX and nitro-BFZ, was characterized in the present work. It was demonstrated that these compounds inhibit the 3’-processing equally or more efficiently than the strand transfer. The influence of the structure of nitro-BFX and nitro-BFZ on their inhibitory activity was studied. The most active integration inhibitors were identified to be 4-nitro- BFZ/BFX containing a methyl group at positions 5 and 7, as well as 5-nitro-BFZ. The described inhibitors also exhibited activity against mutant forms of IN resistant to Raltegravir. The study of the mechanism of IN inhibition by nitro-BFZ and nitro-BFX showed that these compounds prevent the binding of DNA-substrate at the enzyme active site and do not interact with the metal-cofactor ion. The comparison of the pharmacodynamic and pharmacokinetic characteristics of the investigated substances and Raltegravir show promise with respect to further investigations of these compounds as inhibitors of HIV-1 IN.

## Abbreviations

ADME: absorption, distribution, metabolism, and excretion
AIDS: acquired immunodeficiency syndrome
BFX: benzofuroxan
BFZ: benzofurozan
HIV-1: human immunodeficiency virus type 1
IN: integrase
IC50: inhibitor concentration at which the enzyme activity is suppressed by 50 %
C95: inhibitor concentration at which the enzyme activity is suppressed by 95 %
PASS: Prediction of Activity Spectra for Substances software program
QikProp: ADME prediction software program
